# HPV DNA testing and treatment strategies for cervical pre-cancer among women living with HIV in Papua New Guinea: a prospective clinical cohort study

**DOI:** 10.1186/s44263-026-00336-0

**Published:** 2026-10-16

**Authors:** Joyce Sauk, Michaela A Riddell, Joy Tongela, Fredah Koluk, Hari Lialu, Grace Tan, Robert Jones, Arthur Elijah, Josephine Gabuzzi, Paulus Ripa, Pamela J Toliman, Heni Meke, Steven G Badman, Rebecca J Guy, Marion Saville, Angela Kelly-Hanku, Andrew JB Vallely

**Affiliations:** 1 Kirby Institute for Infection and Immunity in Society, UNSW Sydney, Wallace Wurth Building, Kensington, NSW 2052 Australia; 2 Papua New Guinea Institute of Medical Research, New EHP 441 Goroka, Papua New Guinea; 3 Anglicare Papua New Guinea, PO Box 6491, Boroko, 111, NCD, Papua New Guinea; 4 Australian Centre for Prevention of Cervical Cancer, 265 Faraday Street, VIC 3053 Carlton, Australia; 5 Department of Obstetrics and Gynaecology, Port Moresby General Hospital, Taurama Rd,121, NCD, Port Moresby, Papua New Guinea; 6 Western Highlands Provincial Health Authority, Mt Hagen, WHP 281, PO Box 36, Papua, New Guinea

**Keywords:** Cervical cancer, Screening, Prevention, HIV, Papua New Guinea

## Abstract

**Background:**

The World Health Organization recommends human papillomavirus (HPV) testing and same-day treatment for cervical screening among women in the general population in low- and middle-income countries (LMIC). Among women living with HIV, additional triage is recommended due to lower specificity and predictive value of HPV testing alone in this population. We evaluated the performance of HPV DNA testing alone and in combination with alternative triage strategies for the early detection and treatment of cervical pre-cancer among women living with HIV in Papua New Guinea (PNG).

**Methods:**

We did a prospective clinical cohort study among women living with HIV aged 30-59 years attending an HIV treatment and care service. Eligible women self-collected a vaginal specimen for point-of-care HPV DNA testing on the GeneXpert platform. All women testing HPV-positive, and a random sample of HPV-negative women, had enhanced visualisation of the cervix with digital colposcopy (EVA) and provided a clinician-collected cervical specimen for off-site liquid-based cytology. The primary outcome was the clinical performance (sensitivity, specificity, positive and negative predictive value [PPV, NPV]) of HPV testing alone and in combination with alternative triage strategies (EVA, HPV type restriction, modified assay cut-off values) for detection of high-grade squamous intraepithelial lesion (HSIL) or worse.

**Results:**

Between May 3, 2024 and December 12, 2024, we recruited 404 women, of whom 168 (41.6%) tested positive for one or more of 14 oncogenic HPV types. Estimated sensitivity and NPV for HSIL or worse using manufacturer-defined cycle threshold cutoffs was >99%, specificity 63.3% (95%CI: 58.3%-68.3%), and PPV 18.6% (12.6%-24.6%). Restricting screen-positive to one or more of the 8 HPV types of greatest oncogenic potential had comparable sensitivity and NPV (>99%), with specificity 70.2% (65.5%-74.9%) and PPV 22.6% (15.6%-29.6%). HPV 8-type restriction with more stringent assay cut-offs (cycle threshold <33.99) had sensitivity 86.5% (75.3%-97.7%), specificity 80.1% (75.9%-84.3%), PPV 30.7% (21.7%-39.7%), and NPV 98.4% (96.9%-99.9%). EVA provided no additional benefits to HPV testing alone.

**Conclusions:**

Applying HPV 8-type restriction with or without modified manufacturer assay cut-off values to the WHO pre-qualified Xpert HPV test improved clinical performance for detection of HSIL or worse among women living with HIV in PNG.

## Background

Cervical cancer is the fourth most common cancer in women worldwide, with an estimated 661,000 cases and 348,000 related deaths annually [1]. More than 85% of the global burden is experienced by women and their families in low- and middle-income countries (LMIC), where the highly-effective strategies of human papillomavirus (HPV) vaccination, HPV-based cervical screening, and treatment of cervical pre-cancer and cancer, are largely unavailable [2]. Women living with HIV bear a disproportionate burden of cervical pre-cancer and cancer [3, 4] and have an estimated six-fold increased risk of developing invasive disease compared to women in the general population [5]. Papua New Guinea (PNG) has among the highest estimated cervical cancer incidence and mortality rates globally, and the highest rates in the Asia-Pacific region, with incidence 5 times that of Australia (age-standardized rate [ASR] 28.0 vs. 5.2 cases per 100,000), and mortality 14 times greater (19.9 vs. 1.4 cases per 100,000) [1, 2] Cervical cancer is the most common cancer among women in PNG and leads to an estimated 1200–1500 preventable deaths every year [6]. PNG also has one of the fastest growing HIV epidemics in the Western Pacific and experienced a significant surge in HIV diagnoses in 2024/25 with an estimated 30 new infections per day and over 11,000 new infections [7]. The deteriorating situation led the PNG Government to declare a national HIV emergency in June 2025 and to a subsequent tripling of Australian development assistance to strengthen HIV services in the country [7, 8]. The first field trials to demonstrate the clinical performance, safety, acceptability, scalability, and cost-effectiveness of a fully integrated point-of-care HPV self-collect, test, and treat strategy for same-day cervical screening in primary health care settings in a LMIC were carried out in PNG [6, 9–18]. This approach comprises point-of-care HPV DNA testing of self-collected vaginal specimens followed by same-day thermal ablation or gynaecology referral for women who test HPV-positive. It enabled 90% of all high-grade squamous intraepithelial lesions (HSIL) or worse to be detected and treated the same day [6, 16]; was highly acceptable among women, their families, and health workers [17]; can be delivered by trained nursing staff in routine primary health facilities [16]; and is highly cost-effective and efficient compared to earlier screening strategies based on Pap test cytology or visual inspection of the cervix after acetic acid application (VIA) [18]. Following findings from the PNG field trials, the endorsement of HPV screen-and-treat for primary cervical screening by the World Health Organization (WHO) in 2021 [19], and the launch of the WHO global strategy to accelerate the elimination of cervical cancer as a public health problem in 2020 [20], the PNG Technical Working Group on Cervical Cancer, jointly convened by the National Department of Health and the PNG Obstetrics and Gynaecology Society, recommended the introduction and national scale-up of HPV screen-and-treat for women in the general population in December 2021. The success of HPV screen-and-treat in PNG has now led to this strategy being adopted as the primary national screening strategy in Vanuatu, Fiji, Tuvalu, Nauru, Tonga, Samoa, Solomon Islands,Marshall Islands, Kiribati, and Timor-Leste (2022-25) [21–23]. Among women living with HIV, HPV-based screening has been found to have comparable sensitivity but lower specificity compared with women in the general population, raising concerns about possible over-treatment and risks of adverse reproductive health outcomes in this population [24, 25]. Modelling studies have found that the inclusion of an additional triage step among women living with HIV who test HPV-positive could reduce pre-cancer treatments by up to 50% with minimal impact on the effectiveness of screening algorithms to detect and treat cervical pre-cancer [5]. These findings informed the revised WHO guidelines released in 2021 that recommend a HPV screen, triage, and treat approach for women living with HIV in which primary HPV testing is followed either by partial HPV genotyping (HPV 16, 18 with or without HPV 45), cervical cytology, VIA, or colposcopy for triage to guide clinical management and treatment of women who test HPV-positive [19]. A recent systematic review and meta-analysis found that the low specificity of HPV screening algorithms based on the detection of 14 oncogenic HPV types (HPV 16, 18, 31, 33, 35, 39, 45, 51, 52, 56, 58, 59, 66, and 68) could be significantly improved by restricting algorithms to the 8 HPV types of greatest oncogenic potential (HPV 16, 18, 31, 33, 35, 45, 52 or 58), with only minor losses in sensitivity; and that this approach had better overall sensitivity and specificity than triage using either VIA or conventional cytology (Papanicolaou method) among women living with HIV [25]. Additional adaptations, including modified manufacturer cut-off values used to define screen-test positivity, further increased specificity among HPV-positive women living with HIV in South Africa [24]. HPV 8-type restriction, with or without modifications to manufacturer cycle threshold cut-offs, could remove the necessity for a separate triage step in high-burden LMIC settings where an additional clinic visit may be logistically challenging, and for which optimal triage approaches remain unclear [24–27].

Building on these findings and our earlier field research in PNG, in this study we aimed to assess the clinical performance, acceptability, and health systems implementation requirements of point-of-care HPV DNA testing alone and in combination with alternative forms of triage (visualisation with acetic acid and digital colposcopy; HPV type restriction and modified assay cut-off values) for the early detection and treatment of cervical pre-cancer among women living with HIV. In this paper we report the primary study outcome which was the clinical performance evaluation.

## Methods

### Study design and participants

We conducted a non-randomised prospective clinical cohort study among women living with HIV aged 30–59 years attending an HIV treatment and care service at a dedicated nationally accredited facility located in the National Capital District, PNG (Begabari Sexual Health Clinic, Waigani). This age range was selected in accordance with current PNG national screening guidelines [21]. This clinical site was selected because it is one of the largest HIV treatment and care centres in the country and a recognised centre of excellence.

Women aged 30–59 years attending HIV treatment and care services, willing to comply with study procedures, and able to provide written informed consent, were eligible to participate. Women were provided with information about the study during their current clinic visit or contacted by telephone by a member of the clinic team if between scheduled clinic visits. Women who expressed an interest were invited to attend a co-located screening clinic established onsite for the purpose of the study. Women who were pregnant or who had given birth in the past six weeks, or women who had a history of cervical cancer or hysterectomy, were excluded. Women with abnormal laboratory investigations at their most recent HIV treatment and care clinic visit (CD4 less than 200 cells/µL; haemoglobin less than 80 g/L) were excluded. Women who were menstruating at the time of their clinical visit were advised to return for enrolment 1–2 weeks later.

### Procedures

Women were provided with general information about the study on arrival through a 5–10 min group talk (referred to as a *tok save* in PNG Pidgin), given by a member of the clinical research team. A pictorial flipchart developed in our earlier research was used to explain key procedures, such as specimen self-collection and procedures for those with a positive HPV DNA test result. Examples of unused vaginal swabs, specimen collection kits, and vaginal speculums were made available for women to look at and handle. At the end of the talk, women were given an opportunity to ask questions and provided with a copy of the study participant information sheet (in English or PNG Pidgin as preferred). Women interested in participating in the study completed a short eligibility assessment administered by study staff and provided written informed consent. Study staff then carried out a short clinical interview to collect baseline socio-demographic, behavioural, and clinical information using a study-specific case record form.

We used a pictorial guide developed in our earlier field trials to advise women how to collect a mid-cavity vaginal specimen. As per PNG national guidelines and standard operating procedures for point-of-care HPV DNA screening and treatment among women in the general population, we used a flocked swab (FLOQSwab, Copan, Italy) as our preferred specimen collection device. Following agitation of the sample in 5 mL of ThinPrep PreservCyt solution (Hologic, Marlborough, MA, USA), 1 mL of PreservCyt fluid was tested on the rapid fully automated GeneXpert real-time polymerase chain reaction (PCR) platform (Cepheid, Sunnyvale, CA, USA) by a trained laboratory technician (HL) in the clinic, as per manufacturer instructions.

The Xpert HPV Test is a WHO prequalified in-vitro diagnostic assay comprising disposable cartridges that hold the reagents, primers, and probes for the simultaneous detection of 14 oncogenic HPV types (i.e. HPV 16, 18, 31, 33, 35, 39, 45, 51, 52, 56, 58, 59, 66, and 68), which are detected in five fluorescent channels (referred to as channels P1-5), a human reference gene to assess adequacy of the sample (detected in channel P6), and an internal control to verify reagent integrity and processing adequacy. The system monitors the presence of inhibitors in the real-time PCR assay to signal a potentially false negative result. HPV test results are grouped by channel on the GeneXpert platform: HPV 16 (channel P1); HPV 18 or 45 (or both; channel P2); HPV 31, 33, 35, 52, or 58 (or a combination of these types; channel P3); HPV 51 or 59 (or both; channel P4); and HPV 39, 56, 66, or 68 (or a combination of these types; channel P5). The instrument output includes cycle threshold values for each of these channels. The manufacturer has determined cycle threshold cutoff values for each channel to define positivity (for channels P1 and P2, the cutoff value is 40, and for channels P3-P5, it is 38). As per other PCR platforms, lower cycle threshold values on the system are indicative of higher HPV viral load in the sample (less processing cycles are required to detect HPV DNA), and higher cycle threshold values are associated with lower HPV viral load (greater number of processing cycles required to detect HPV DNA).

All women with a positive HPV DNA test were counselled about their result and offered a pelvic examination in which a trained nurse (JT, FK, supported by medical practitioner, JS) carried out enhanced visual assessment of the cervix with acetic acid using a portable handheld digital colposcope (EVA Colposcope, MobileODT, Israel) to assess if referral to a gynaecologist (RJ, AE) was indicated due to the presence of a cervical lesion suspicious of cancer or other abnormalities such as a cervical polyp. Digital images of the cervix were captured under white light and green filter illumination and uploaded to an encrypted cloud-based server. Staff recorded clinical examination findings in a study-specific case record form and as annotations and brief notes accompanying digital image files. All digital image files were reviewed by JS.

All women who tested HPV-positive and agreed to have a pelvic examination were asked to provide a cervical specimen collected by a clinician, before application of acetic acid, for assessment of the study primary outcome. Cervical specimens were collected using a Cervex-Brush Combi (Rovers Medical Devices, Oss, Netherlands), which enabled simultaneous collection of cells of the ectocervix, endocervix, and transformation zone in a single sample. Following collection, specimens were immediately placed in 20 mL of PreservCyt and stored onsite at 4 °C before shipment to VCS Pathology at the Australia Centre for the Prevention of Cervical Cancer (ACPCC, Melbourne, VIC,

Australia), a laboratory accredited by the National Association of Testing Authorities–Royal College of Pathologists Australia. At ACPCC, liquid-based cytology (LBC) was carried out in accordance with established laboratory procedures. All specimens were independently read by two separate teams consisting of scientists and pathologists (including GT, overseen by MS), who were masked to HPV DNA test results and the other team’s assessment results. When both teams agreed on a diagnosis of HSIL or worse, no further investigation was instituted, and a final diagnosis was recorded. If the assessment differed and one team assessed the case to be possible HSIL or worse, p16/Ki-67 dual stain cytology (CINTec PLUS Cytology, Roche Diagnostics, Rotkreuz, Switzerland) was carried out and the result used to make a final diagnosis.

A random systematic sample of 120 HPV-negative women were also asked to provide a cervical specimen collected by a clinician for LBC. Selection was based on study numbers allocated sequentially at enrolment from a preprinted study register. Based on our a priori assumption that around 30–40% of women living with HIV would have a positive HPV test result and around 60–70% of women would be HPV negative, one in three study numbers in the register were systematically marked before the start of the study to indicate which women should be invited to provide a specimen. If a woman allocated a marked study number from the register was found to test positive for HPV, or an HPV-negative woman declined to provide a specimen, clinic staff were instructed to ask the next HPV-negative woman to do so. All women with a negative HPV test were counselled regarding their test result and advised to return to the clinic for HPV-based screening in 3 years. HPV-negative women were not routinely offered a pelvic examination, unless indicated for clinical reasons.

Women with a positive HPV test result were advised to return to the clinic to discuss their LBC results in three months or earlier if contacted directly by staff. Women who had HSIL on LBC were counselled about their test result and offered a repeat pelvic examination to assess their eligibility for thermal ablation. Women with a healthy cervix on naked eye examination after application of acetic acid, and who had a cervical transformation zone visible in its entirety, were offered same-day ablation, in which the entire transformation zone was treated using one to five applications of the thermal ablation probe as required. If an abnormality was seen on examination, such as a suspected cervical cancer or a cervical polyp, or the transformation zone was not visible in its entirety, the woman was advised that referral for specialist review was necessary, and an appointment was made for her to attend the gynaecology outpatient clinic at Port Moresby General Hospital. Pelvic examination, eligibility assessment, and same-day thermal ablation or gynaecology referral were all carried out by a trained nurse and conducted in accordance with study specific standard operating procedures.

Women who had LBC findings worse than HSIL (squamous cell carcinoma [SCC], adenocarcinoma in situ [AIS]) were contacted by telephone immediately results were available to the clinic team and were asked to attend the study clinic urgently, when they were counselled regarding findings and urgent (same week) referral to the gynaecology outpatient clinic at Port Moresby General Hospital made.

Women with LBC findings less than HSIL (low-grade squamous intraepithelial lesion [LSIL], atypical squamous cells of undetermined significance [ASCUS], normal examination) were counselled about their test results at their three-month clinic visit and advised that no treatment was indicated at this point.

All women who tested HPV-positive were advised to return to the clinic in 12 months’ time for repeat HPV-based screening.

### Outcomes

The primary outcome of the study was the clinical performance (i.e. sensitivity, specificity, positive predictive value [PPV], and negative predictive value [NPV]) of point-of-care HPV DNA testing alone and in combination with alternative forms of triage for the detection of cervical HSIL or worse among women living with HIV in PNG. Triage options evaluated were enhanced visual assessment of the cervix with acetic acid using digital colposcopy (EVA); HPV partial type restriction (HPV 16, 18, or 45); HPV 8-type restriction (HPV 16, 18, 31, 33, 35, 45, 52 or 58); and varying assay cycle threshold (Ct) value cutoffs. Secondary outcomes were client and provider acceptability, and health system implementation requirements. These will be reported in a separate manuscript.

As in our earlier field trials in PNG [6, 16], and studies conducted in other LMIC settings, [28–30] we used LBC as the reference standard against which to evaluate our screening algorithm for two reasons. First, outside of pure research settings, it is ethically questionable to use cervical biopsy to assess women who test HPV-negative at their primary screening visit [31] and therefore, histology cannot be used as the reference standard for measuring the specificity of real-world screening algorithms. Second, although colposcopy-guided biopsy for a histological diagnosis among HPV-positive women is the preferred standard in high-income settings, this approach cannot be implemented in routine health-care settings in many LMIC, including PNG.

### Statistical analysis

Based on published data from other settings [32–37], we conservatively estimated that the prevalence of oncogenic HPV infection among women living with HIV would be around 30% and that around 10% of those screened would have HSIL or worse. We estimated that a study among 400 women living with HIV would therefore enable us to identify 120 HPV-positive women (30% of total) and around 40 women with HSIL or worse (10% of total). To estimate the performance of HPV-based screening for the detection of HSIL or worse, we collected cervical specimens from all HPV-positive women (120 women of 400 enrolled) and a similar number of randomly selected HPV-negative women. We calculated that this sample size would be sufficient to estimate the clinical performance characteristics (sensitivity, specificity, positive and negative predictive values) of our HPV-based screening algorithm with around 5% precision (i.e. specificity of 90.0% would be estimated with 95% confidence interval [CI] of 85.4%-94.6%). All sample size calaculations and statistical analyses were done using Stata (version 18.0).

## Results

Between May 3, 2024 and December 12, 2024, a total of 404 women were recruited, all of whom had previously attended for HIV treatment and care services at the participating clinic. Clinical follow-up was completed 25 April 2025; data entry, validation and statistical analyses were completed 15 October 2025.

At enrolment, 168 (41.6%) women tested positive for one or more oncogenic HPV type and 236 (58.4%) had a negative HPV test (Table 1). HPV 16 was prevalent in 29 (7.2%) women, HPV 18 or HPV 45 in 30 (7.4%) women, and other HPV types in 138 (34.2%) women. There were no substantial differences in sociodemographic characteristics of women by HPV test result at enrolment (Table 1). Sociodemographic data among HPV-negative women who consented to have a pelvic examination and to provide an LBC specimen (see below) were similar to those among all 236 HPV-negative women (data not shown).

**Table 1:** Selected baseline socio-demographic, behavioural and clinical characteristics of study participants by HPV status (*N* = 404)

	All *n* = 404	HPV negative *n* = 236; 58.4%	HPV positive *n* = 168; 41.6%	*p* value
**Age (median, IQR)**	38 (32-43)	38 (33-44)	36 (32-42)	**p=0.0193**
**Marital status**	*n* = 404	*n* = 236	*n* = 168	
- Married (% of total)	214 (53.0)	132 (55.9)	82 (48.8)	*p* = 0.1593
- Single (% of total)	40 (9.9%)	20 (8.5)	20 (11.9)	*p* = 0.2603
- Widowed (% of total)	40 (9.9%)	27 (11.4)	13 (7.7)	*p* = 0.2195
- Separated/divorced (% of total)	110 (27.2)	57 (24.2)	53 (31.5)	*p* = 0.1047
**Education level - highest achieved**	*n* = 399	*n* = 235	*n* = 164	
- Did not go to school	78 (19.6)	45 (19.2)	33 (20.1)	*p* = 0.8238
- Completed Grade 1–6	102 (25.6)	63 (26.8)	39 (23.8)	*p* = 0.4997
- Completed Grade 7–10	145 (36.3)	90 (38.3)	55 (33.5)	*p* = 0.3273
- Completed Grade 11, 12	30 (7.5)	13 (5.5)	17 (10.4)	*p* = 0.0681
- Completed university, vocational or technical college	44 (11.0)	24 (10.2)	20 (12.2)	*p* = 0.5307
**Current employment**	*n* = 400	*n* = 232	*n* = 168	
- Not working	12 (3.0)	7 (3.0)	5 (3.0)	*p* = 1.0000
- Subsistence farming	4 (1.0)	3 (1.3)	1 (0.6)	*p* = 0.4892
- Household duties	148 (37.0)	88 (37.9)	60 (35.7)	*p* = 0.6532
- Formal salaried employment	77 (19.2)	46 (19.8)	31 (18.4)	*p* = 0.7261
- Marketing (selling home grown produce, other goods at market)	123 (30.8)	71 (30.6)	52 (31.0)	*p* = 0.9319
- Self-employed business	30 (7.5)	15 (6.5)	15 (8.9)	*p* = 0.3692
- Student	6 (1.5)	2 (0.9)	4 (2.4)	*p* = 0.2283
**Religion**	*n* = 403	*n* = 235	*n* = 168	
- Anglican	4 (1.0)	3 (1.3)	1 (0.6)	*p* = 0.4885
- Catholic	55 (13.6)	30 (12.8)	25 (14.9)	*p* = 0.5457
- Lutheran	28 (7.0)	16 (6.8)	12 (7.1)	*p* = 0.9070
- United Church	19 (4.7)	11 (4.7)	8 (4.8)	*p* = 0.9629
- SDA	118 (29.3)	67 (28.5)	51 (30.4)	*p* = 0.6798
- Pentecostal	33 (8.2)	20 (8.5)	13 (7.7)	*p* = 0.7727
- Other	146 (36.2)	88 (37.4)	58 (34.5)	*p* = 0.5508
**HPV vaccine**	*n* = 399	*n* = 235	*n* = 164	
- Previously received HPV vaccine	0	0	0	-

Of 193 women who had a pelvic examination and digital colposcopy at enrolment, 36 (18.7%) were HPV-negative and 157 (81.3%) HPV-positive. The most common examination findings were normal cervical appearance (*n* = 183), vaginal discharge with inflammation of the cervix (*n* = 15); bulging or inflamed cervix (*n* = 9). Among women who tested HPV-positive, 7.6% (12 of 157) had abnormal findings on examination requiring gynaecology referral, among whom one woman was subsequently found to have SCC on LBC, one had LSIL, six had negative LBC, one had an unsatisfactory specimen, and three refused specimen collection. Among HPV-negative women, there were no clinical findings requiring gynaecology referral.

Cervical specimens were provided by 87.5% (147 of 168) HPV-positive women. Among HPV-negative women, 40 women (33.3% of 120 target) provided a cervical specimen for LBC. Of the total of 187 cervical specimens collected at enrolment, 24 (12.8%) were considered unsatisfactory for LBC due to blood staining or low cellularity, with similar proportions among HPV-positive women (18/147, 12.2%) and HPV-negative women (6/40, 15.0%; *p* = 0.639). Accordingly, 163 LBC specimens from 34 HPV-negative and 129 HPV-positive women were examined (Table 2).

**Table 2:** Liquid-based cytology findings by HPV status (*N* = 163)^#^

	HPV negative	HPV positive (any)	HPV16 positive (all)*	HPV18 or 45 positive (all)*	HPV Other positive (all)*	HPV16,18,45 positive (only)^	HPV31,33,35,52,58,51,59, 39,56,66,68 positive (only)^	HPV16,18,45,31,33,35,52, 58 positive (only)^	HPV51,59, 39,56,66,68 positive (only)^
		Any of Xpert channel P1-5 positive	Xpert channel P1 positive +/- other channels	Xpert channel P2 positive +/- other channels	Any of Xpert channel P3-5 positive +/- other channels	Xpert channel P1 or P2 positive only	Xpert channel P3, P4 or P5 positive only	Xpert channel P1, P2, or P3 positive only	Xpert channel P4 or P5 positive only
Negative	33	71	12	16	56	25	46	54	13
ASCUS or LSIL	1	34	6	4	28	10	24	28	7
HSIL	0	20	5	4	15	7	14	20	0
AIS	0	0	0	0	0	0	0	0	0
SCC	0	4	2	1	2	2	2	4	0
**Total**	**34**	**129**	**25**	**25**	**101**	**44**	**86**	**106**	**20**

ASCUS=atypical squamous cells of undetermined significance, LSIL = low-grade squamous intraepithelial lesion, HSIL=high-grade squamous intraepithelial lesion, AIS=adenocarcinoma in situ, SCC=squamous cell carcinoma

^#^ 187 LBC specimens were assessed of which 163 (87.2%) were satisfactory and 24/187 (12.8%) were unsatisfactory (among HPV+ unsat = 12.2%, among HPV- unsat = 15.0%)

* women who had a positive test for HPV type of interest irrespective of whether also positive for other HPV types

^ women who had a positive test for specified HPV types only

Among 129 HPV-positive women with a satisfactory specimen for LBC, 25 (19.4%) tested positive for HPV 16; 25 (19.4%) tested positive for HPV 18, HPV 45, or both; and 101 (78.3%) tested positive for other HPV types. The prevalence of HSIL or worse was higher among women with HPV 16 (7 [28.0%] of 25) than among women with HPV 18 or HPV 45 (5 [20·0%] of 25; *p* = 0.512), or other HPV types (17 [16.8%] of 101; *p* = 0.203). When analysis was restricted by positivity on certain GeneXpert channels only, the prevalence of HSIL or worse was highest among women with HPV 16, 18, 45, 31, 33, 35, 52, or 58 only (channel P1, P2, or P3 positive; 24 [22.6%] of 106), than among those with HPV 16, 18 or 45 only (channel P1 or P2 positive; 9 [20.5%] of 44), or those with HPV 31, 33, 35, 52, 58, 51, 59, 39, 56, 66, or 68 only (channel P3, P4, or P5 positive (16 [18.6%] of 86). There were no cases of HSIL or worse among women with HPV 51, 59, 39, 56, 66, or 68 only (channel P4 or P5 positive only). Among the 34 HPV-negative women with a satisfactory specimen for LBC, there were no cases of HSIL or worse detected.

Among HPV-positive women subsequently found to have HSIL or worse, three of four SCC cases (75%), and all the 20 HSIL cases (100%), had unremarkable pelvic examination and digital colposcopy findings; and one SCC (25%) case was referred for gynaecology review due to a suspicious cervical lesion on examination. It was not therefore possible to further evaluate the performance of enhanced visual assessment by digital colposcopy as a triage step in this setting.

Estimates of high-grade disease among the 163 HPV-positive women and HPV-negative women who provided a satisfactory specimen for LBC were used to estimate the overall prevalence of high-grade disease in the full study cohort of 404 women and to calculate estimates of clinical performance characteristics using different HPV type restrictions and assay cycle threshold cutoffs (Table 3).

**Table 3:** Estimated performance of point-of-care Xpert HPV testing of self-collected vaginal specimens for detection of HSIL or worse among women living with HIV in Papua New Guinea (*n* = 404)

	High grade disease		Percentage %			
	Positive *n*	Negative *n*	Sensitivity (95% CI)	Specificity (95% CI)	PPV (95% CI)	NPV (95% CI)
**Algorithm 1**						
Any HPV (Xpert P1-5) positive	31	137	100%	63.3% (58.3, 68.3)	18.6% (12.6, 24.6)	100%
HPV negative	0	236				
**Algorithm 2**						
HPV16,18,45 (Xpert P1-2) positive	12	46	36.0% (19.3, 52.7)	87.8% (84.4, 91.2)	20.5% (7.4, 33.6)	94.0% (91.5, 96.5)
HPV negative plus HPV Other (Xpert P3-5) positive	21	327				
**Algorithm 3**						
Any of HPV16,18,45,31,33,35,52,58 (Xpert P1-3) positive	32	111	100%	70.2% (65.5, 74.9)	22.6% (15.6, 29.6)	100.0%
HPV negative plus Any of HPV51,59,39,56,66,68 (Xpert P4-5) positive	0	261				
**Algorithm** **4**						
Any of HPV16,18,45,31,33,35,52,58 (Xpert P1-3) positive with Ct *≤* 29.99	26	44	72.2% (57.3, 87.1)	88.0% (84.6, 91.4)	37.2% (25.6, 48.8)	97.0% (95.1, 98.9)
HPV negative plus HPV16,18,45,31,33,35,52,58 (Xpert P1-3) positive with Ct *≥* 30.0 plus Any of HPV51,59,39,56,66,68 (Xpert P4-5) positive	10	324				
**Algorithm 5**						
Any of HPV16,18,45,31,33,35,52,58 (Xpert P1-3) positive with Ct *≤* 33.99	32	73	86.5% (75.3, 97.7)	80.1% (75.9, 84.3)	30.7% (21.7, 39.7)	98.4% (96.9, 99.9)
HPV negative plus HPV16,18,45,31,33,35,52,58 (Xpert P1-3) positive with Ct *≥* 34.0 plus Any of HPV51,59,39,56,66,68 (Xpert P4-5) positive	5	294				

Algorithm 4 would treat 26+44/404 (17.3%) of all WLHIV screened and have high PPV but low SENS; Algorithm 5 would treat 32+73/404 (26.0%) of all WLHIV screened but with most favourable SENS, PPV, NPV

The estimated sensitivity and NPV for the detection of HSIL or worse using manufacturer-defined cycle threshold cutoffs for all five channels was greater than 99% but had sub-optimal specificity (63.3%; 95%CI: 58.3%-68.3%) and PPV (18.6%; 12.6%-24.6%) (algorithm 1, Table 3). Restricting the definition of clinically relevant HPV positive to women with any of HPV 16, 18 or 45 only (channel P1 or P2 positive only; algorithm 2) led to a substantial reduction in estimated sensitivity (36.0% vs.100%), increased specificity (87.8% vs. 63.3%), and comparable PPV and NPV compared with algorithm 1. Broadening the definition of clinically relevant HPV positive to include women with any of HPV 16, 18, 45, 31, 33, 35, 52, or 58 (channel P1, P2, or P3 positive only; algorithm 3) increased estimated specificity (70.2% vs. 63.3%) and PPV (22.6% vs. 18.6%) and had comparable sensitivity (> 99%) and NPV (> 99%) to algorithm 1. Adopting alternative cycle threshold cutoffs (algorithms 4 and 5, Table 3) led to an increase in estimated specificity and PPV, but lower sensitivity and NPV compared with algorithms 1 or 3. Restricting clinically relevant HPV positivity to channel P1, P2, or P3 positive at cycle threshold of less than 33.99 only (algorithm 5), had high estimated sensitivity (86.5%; 75.3%-97.7%), specificity (80.1%; 75.9%-84.3%), PPV (30.7%; 21.7%-39.7%) and NPV (98.4%; 96.9%-99.9%).

All women with HSIL or worse (24 of 404 women recruited, 5.9%) were contacted by mobile phone or via community networks and asked to return to receive their LBC results and to discuss further treatment. Three of the four SCC cases (75%) attended specialist gynaecology referral as recommended by the clinical team, and 14 of the 20 women with HSIL (70%) completed a repeat pelvic examination and were treated by thermal ablation at the participating study clinic. One woman with HSIL re-located to another province following study enrolment but was successfully followed up and treated at her local clinic. Multiple contact attempts and reminders were necessary to encourage completion of clinical follow-up and treatment. No moderate or serious adverse events following thermal ablation were reported.

## Discussion

We did a prospective clinical cohort study to evaluate point-of-care HPV DNA testing plus alternative forms of triage for the detection of underlying cervical pre-cancer among women living with HIV in Papua New Guinea. Applying an HPV 8-type restriction with or without alternative assay cycle threshold cutoffs to a WHO prequalified HPV DNA test led to substantial improvements in clinical performance characteristics of the assay. Enhanced visual assessment of the cervix using a mobile digital colposcope provided no additional benefits to HPV DNA testing alone in this setting.

Our findings build directly on an earlier study among 535 women living with HIV in South Africa [24] that recommended HPV 8-type restriction in combination with modified assay cutoffs and specifically, that individual cycle threshold values be entered into a calculator programmed with logistic regression parameters to categorise HPV test results as screen-positive or screen-negative. Our approach, based on HPV 8-type restriction only (Xpert P1-3 positive; algorithm 3), or a combination of HPV 8-type restriction plus a single cutoff value (Xpert P1-3 positive plus Ct < 33.99; algorithm 5), had comparable benefits to the South Africa study in terms of improved clinical performance [24]. However, algorithm 3 or 5 would be much more straightforward and operationally feasible to implement among women living with HIV in resource-constrained LMIC health systems because no additional equipment and only minimal extra staff training would be required.

WHO guidelines currently recommend HPV-based screen, triage and treat over HPV screen-and-treat for women living with HIV in LMIC, but uncertainty about choice of an optimal triage step and concerns regarding treatment completion rates and loss to clinical follow-up associated with multi-visit screening algorithms have re-focussed attention on HPV screen-and-treat as a pragmatic alternative [24–27, 31]. Triage by VIA has previously been endorsed by the WHO [19]. However, as noted in a recent systematic review and meta-analysis of screening strategies among women living with HIV,^25^ VIA triage following primary HPV DNA testing is associated with a substantial reduction in overall sensitivity to detect underlying cervical pre-cancer compared to HPV DNA testing alone [25]. A recent randomised controlled non-inferiority trial among 433 women living with HIV in India conducted the first head-to-head comparison of HPV screen-and-treat (arm 1) and HPV screen, triage and treat (arm 2), using VIA as the clinical triage test [27]. Visual assessment was carried out by highly-skilled nurses who had been performing VIA in participating health facilities for more than 10 years. Detection of cervical intraepithelial neoplasia grade 2 (CIN2) or worse was similar by trial arm (16.7% vs. 13.3% in arms 1 and 2, respectively). HPV clearance at 12 months was higher among women in arm 1 compared to arm 2 (56.6% vs. 41.4% *p* = 0.004). A prospective cohort study among 1478 women living with HIV in Botswana found that VIA triage after HPV 8-type restriction improved overall specificity but reduced sensitivity from 87.8% (95%CI: 80.8%-92.6%) to 56.2% (47.2%-64.8%) to detect CIN2 or worse [38].

The poor performance of naked eye VIA for clinical triage has led to the development of alternative strategies based on enhanced visual assessment (EVA), where VIA is augmented by digital colposcopy; and most recently, automated visual evaluation (AVE), where cervical images are analysed using artificial-intelligence (AI) tools. We found that EVA using a portable digital colposcope had low sensitivity for clinical triage among women living with HIV in PNG, detecting only one of 24 cases of HSIL or worse among HPV-positive women. Visual assessments were conducted by experienced nursing staff who attended a nationally accredited in-country training and clinical mentorship course in HPV-based screening and treatment prior to the study and received clinical support from an experienced onsite sexual health physician following the start of enrolment. Offsite review of digital images by an experienced gynaecologist might have improved the performance of digital colposcopy in our study. However, a recent study in Kenya found that digital colposcopy performed by non-physician providers with image review by offsite expert colposcopists had a sensitivity of 21.4% to 35.7% for the detection of CIN2 or worse among HPV-positive women living with HIV, despite 70.9% of digital images being rated as high and 26.6% of medium quality [39].

A study among 24,447 women attending cervical screening services in Malawi, Rwanda, Senegal, Zambia, and Zimbabwe found that AVE, in which images of the cervix after application of acetic acid were captured on a mobile phone and analysed by an onboard AVE app using AI-based algorithms, had greater sensitivity for CIN2 or worse than VIA alone, or VIA plus digital colposcopy, (60.1%, 42.9%, 37.5%, respectively) [40]. Among 9559 (of 24,447, 39.1%) of women living with HIV, AVE had sensitivity 60.8% (95%CI: 54.9%-66.4%) and specificity 79.1% (78.1%-80.1%) for CIN2 or worse. The performance of AVE for clinical triage following HPV DNA testing among women living with HIV was not assessed [40]. A trial of alternative screen, triage and treat strategies among 5000 women living HIV that compares HPV DNA testing, HPV 8-type restriction, VIA, digital colposcopy, p16/Ki-67 dual stain cytology, and E6 and E7 oncoprotein testing, is ongoing in Rwanda [26, 41].

Our study had several possible limitations. Our decision to estimate clinical performance by use of data from all HPV-positive women and a subpopulation of HPV-negative women might have led to underestimation or overestimation of the true clinical performance of the screening algorithms under consideration. There is a theoretical possibility that our systematic random sampling strategy among HPV-negative women might not have been fully adhered to in practice and could have led to selection bias. For example, women of a certain age or educational background might have been more likely to consent to pelvic examination and LBC collection. We believe this risk of bias to be low because sociodemographic characteristics among HPV-negative women who provided an LBC specimen were similar to those among all HPV-negative women. Completion of pelvic examination and provision of LBC specimens among HPV-negative women was also lower than anticipated (40 of 120 target) but as a proportion of all HPV-negative women in the study, was comparable to our earlier field trial (40 of 236, 16.6% vs. 551 of 3638, 15.1%). We experienced difficulties achieving optimal treatment completion and clinic re-attendance rates when implementing a multi-visit screening model in this setting, despite providing reminders via mobile phone and community visits; and scheduling follow-up appointments to coincide with routine HIV treatment and care visits. In contrast, our earlier trial of same-day HPV screen-and-treat among women in the general population achieved 93% same-day treatment completion, with a further 6.5% of HPV-positive women referred for gynaecological review, of whom 88% attended [16]. Comparable treatment completion and referral rates are being achieved during the ongoing scale-up of HPV screen-and-treat in PNG, and it is highly likely such rates could also be achieved among women living with HIV if same-day screen-and-treat approaches were adopted.

To our knowledge, this is the first evaluation conducted in the Western Pacific and has important implications for future policy and practice in PNG and other LMIC in the region. In committing to equitable and inclusive national cervical cancer elimination programs where no woman is left behind, and in the absence of a suitable triage step, policy makers and clinicians could consider the pragmatic decision to implement a modified HPV screen-and-treat strategy based on HPV 8-type restriction with or without alternative assay cycle threshold cutoffs for women living with HIV. In PNG, adopting algorithm 3 (HPV 8-type restriction only) would mean offering treatment to around 35% of women screened (143 of 404; 35.4%) with PPV 22.6% (Table 3). Algorithm 5 (HPV 8-type restriction plus Ct < 33.99) would mean treating around 26% of women screened (105 of 404; 26.0%) with PPV 30.7%. In a setting where HPV positivity among women living with HIV is 42% and treating all HPV-positive women would mean around 81% of women were treated unnecessarily (algorithm 1, PPV 18.6%; Table 3), algorithms 3 and 5 represent highly attractive options. In addition, adoption of modified screen-and-treat approaches would mean the majority of women requiring treatment could be treated the same day. Phased introduction and scale-up, supported by rigorous clinical performance monitoring (e.g. periodic evaluation of PPV in practice based on collection of LBC specimens from HPV-positive women), would significantly advance cervical cancer elimination efforts among women living with HIV who experience among the highest burden of cervical pre-cancer and cancer globally.

## Conclusions

Applying HPV 8-type restriction with or without modified manufacturer assay cut-off values to the WHO pre-qualified Xpert HPV test improved clinical performance for the detection of HSIL or worse among women living with HIV in Papau New Guinea. This modified screen-and-treat approach could be delivered without additional hardware or software requirements and with minimal additional investment in staff training. It represents a pragmatic solution to cervical screening in LMIC settings where multi-visit screening algorithms have previously been associated with poor treatment completion rates.

## Data Availability

Data from this study are available from the Kirby Institute UNSW Sydney / Papua New Guinea Institute of Medical Research collaborative group upon request, and with the permission of responsible research ethics committees in Papua New Guinea and Australia.
